# Supplementary material for: Native globular ferritin nanopore sensor
Source: Nat Commun. 2025 Jun 6;16:5268. doi: 10.1038/s41467-025-60322-2 (PMC12144159; doi:10.1038/s41467-025-60322-2)
Supplement: Supplementary file 1 — Supplementary Information [file 41467_2025_60322_MOESM1_ESM.pdf]

Supplementary Information

Native Globular Ferritin Nanopore Sensor

Yun-Dong Yin[1,2#], Yu-Wei Zhang[1#], Xi-Tong Song[1#], Jun Hu[1], Yu-Heng Chen[1], Wen-Chuan Lai[1], Ya-Fei Li[1], and Zhi-Yuan Gu[1*]

[1]State Key Laboratory of Microbial Technology, Jiangsu Collaborative Innovation Center of Biomedical Functional Materials, Jiangsu Key Laboratory of New Power Batteries, College of Chemistry and Materials Science, Nanjing Normal University, Nanjing, China, 210023.

[2]College of Life Sciences, Nanjing Normal University, Nanjing, China, 210023.

*E-mail: guzhiyuan@njnu.edu.cn

[#] Yun-Dong Yin, Yu-Wei Zhang, and Xi-Tong Song contributed equally.

Supplementary Tables and Figures

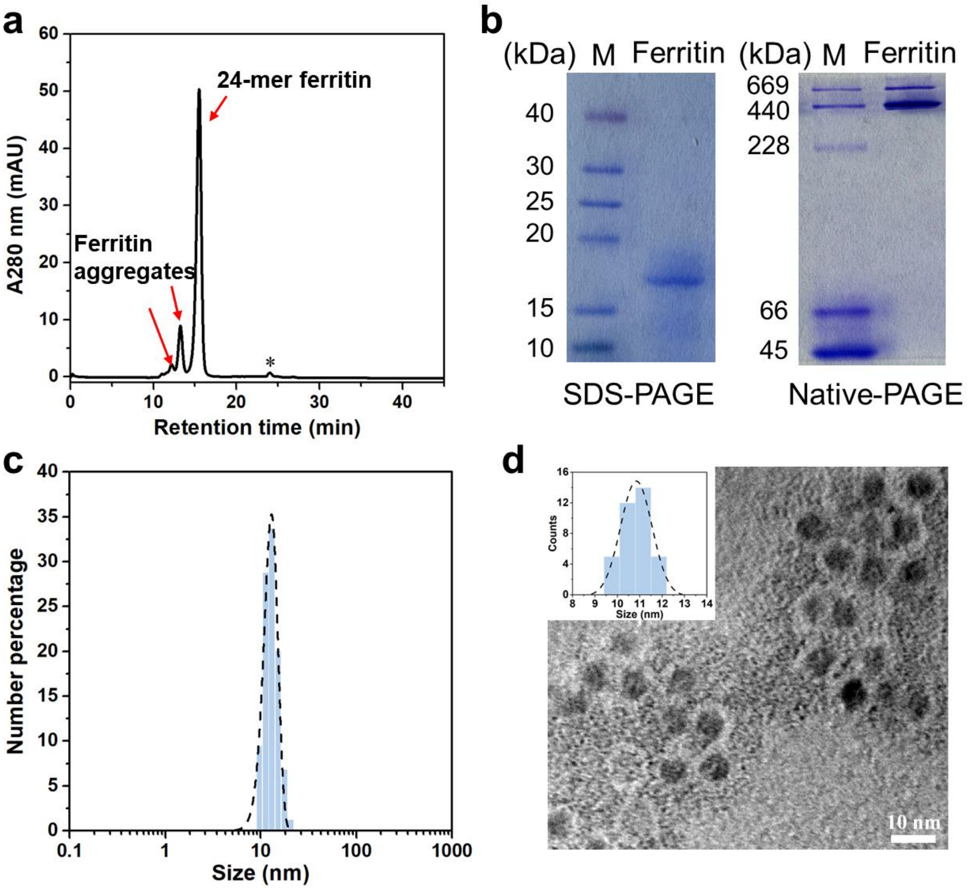

Supplementary Figure 1. Characterization for horse spleen ferritin sample. (a) SEC analysis for ferritin. The main peak at 15.5 minutes represents the monomer ferritin, and the small peaks at 12 and 13 minutes respectively represent the ferritin aggregates, which has been reported previously.[1] The minor peak marked by * may be caused by salts, which was also observed in previous works.[2, 3] There are no other peaks, indicating good purity of the ferritin sample. (b) SDS- and Native-PAGE analysis for ferritin. M: marker, Ferritin: ferritin sample. The SDS-PAGE and Native-PAGE are carried out with 12% gel at 120 V and with 15% gel at 150 V, respectively. In the SDS-PAGE experiment, only a single band between 15 kDa and 20 kDa was observed, with no obvious bands above 20 kDa detected, indicating that the ferritin sample predominantly consists of L-chains which is consistent with previously works.[4] In Native-PAGE experiment, the intact 24-mer ferritin band is observed at ~440 kDa. The band corresponding higher molecular weight (it doesn't mean ~669 kDa but have not been totally expended) means the ferritin aggregates,[5] which is consistent with the SEC experiments in (a). (c-d) DLS analysis and stained HTREM images for ferritin. Insertion is the

size statistic for ferritin. The DLS and HRTEM experiments verify the intact spherical structure (~11 nm) of ferritin sample.[6]

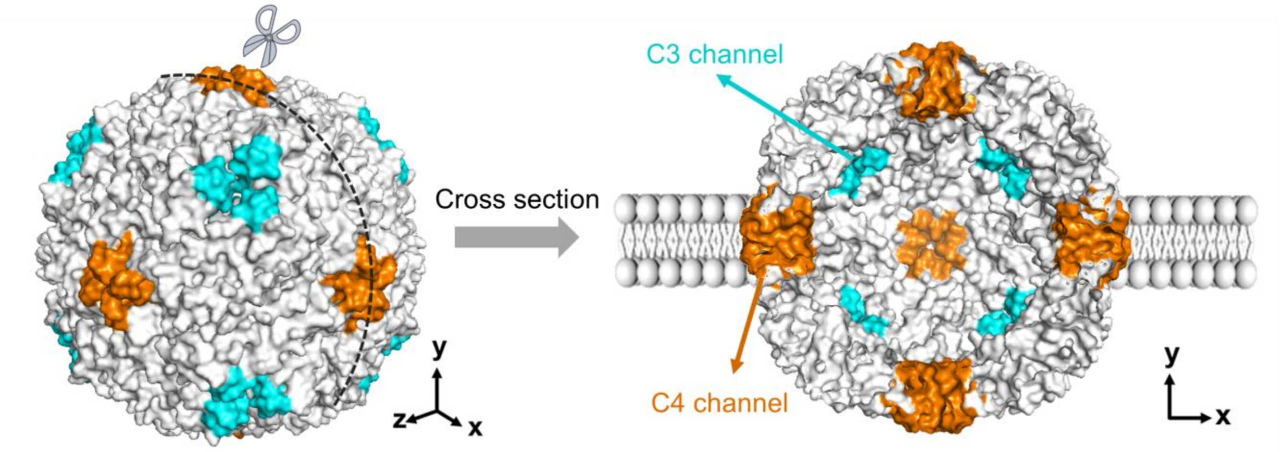

Supplementary Figure 2. Ferritin surface and cross-sectional diagram. All the eight C3 channels and six C4 channels are marked in blue and orange, respectively. Lipid bilayer is located on the XZ plane, with one C4 channel and four C3 channels on each side.

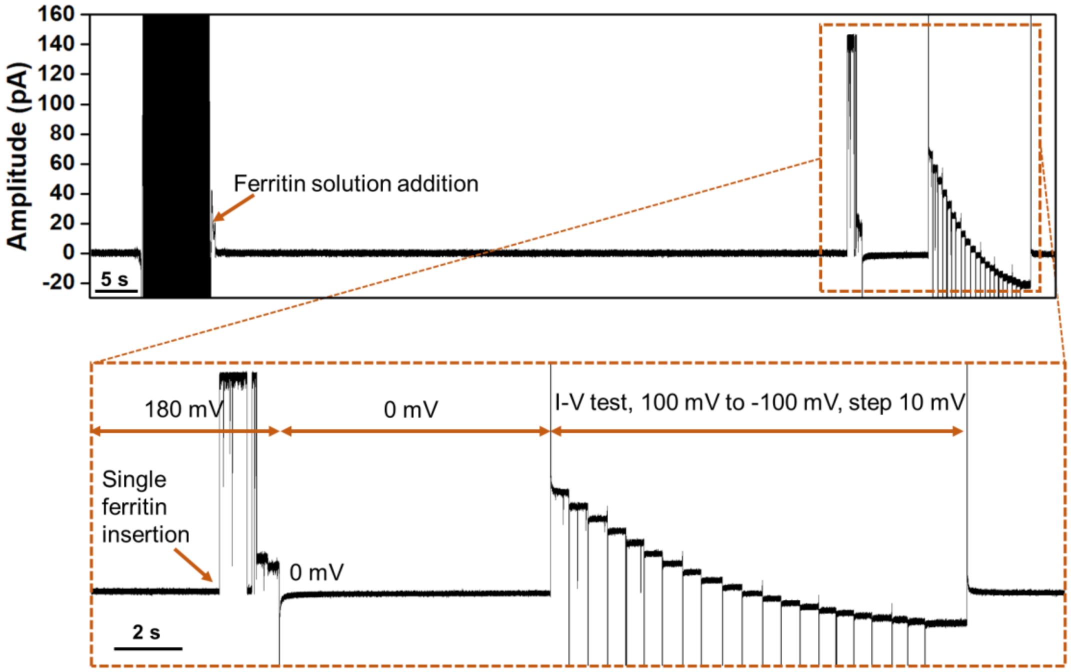

Supplementary Figure 3. Typical current trace of single ferritin inserting into the lipid bilayer at +180 mV. The current change from 0 to ~150 pA represents the insertion of ferritin. The 0 mV bias is applied to ensure the correct ionic current without baseline shift. The I-V test is carried out by regulating voltage from +100 to -100 mV. The electrical recording is carried out in 1 M KCl buffer at pH 7.4.

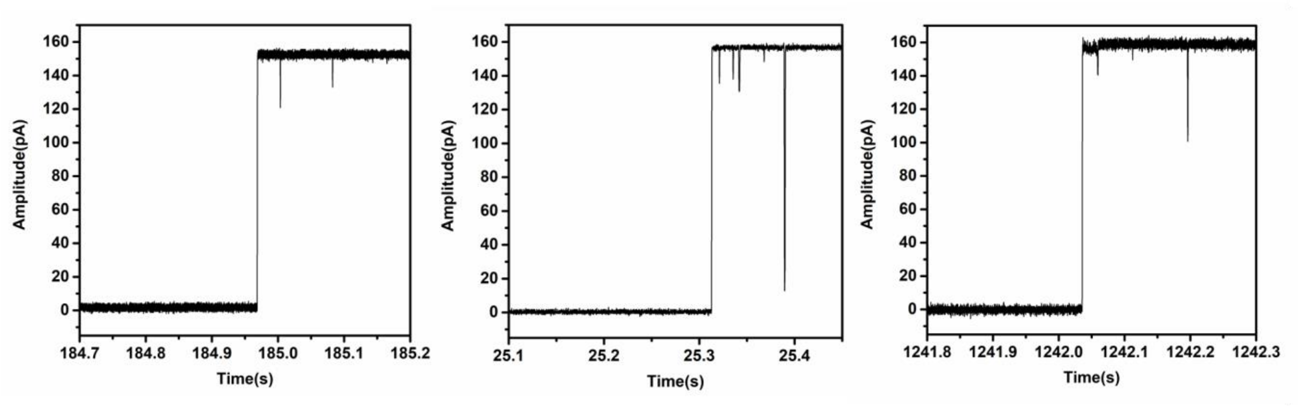

Supplementary Figure 4. Three independent electrical recordings of ferritin nanopore forming. All the experiments are carried out at +180 mV in 1 M KCl buffer at pH 7.4.

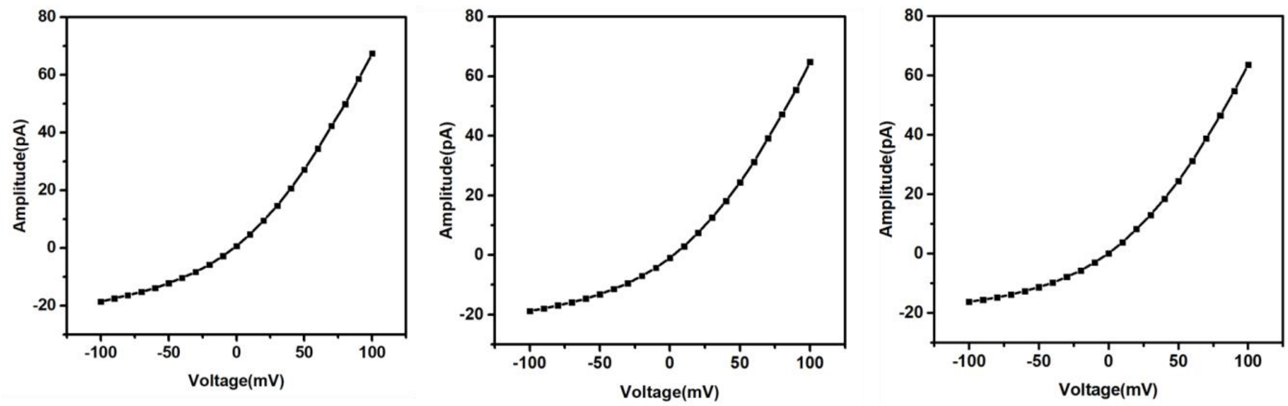

Supplementary Figure 5. Independent I-V curves from three ferritin nanopores. All the experiments are carried out in 1 M KCl buffer at pH 7.4.

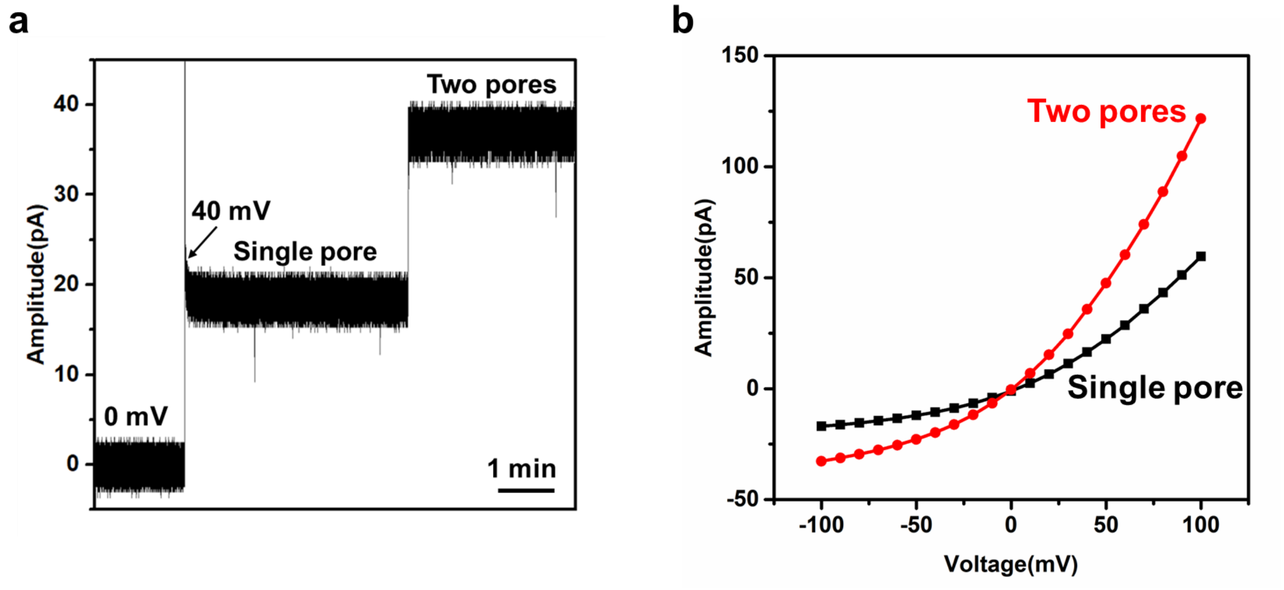

Supplementary Figure 6. (a) Current trace of single and two ferritin nanopores. The first pore is beforehand inserted. The second pore is inserted in the period of electrical recording. (b) I-V curves of single and two ferritin nanopores. All the experiments are carried out in 1 M KCl buffer (pH 7.4).

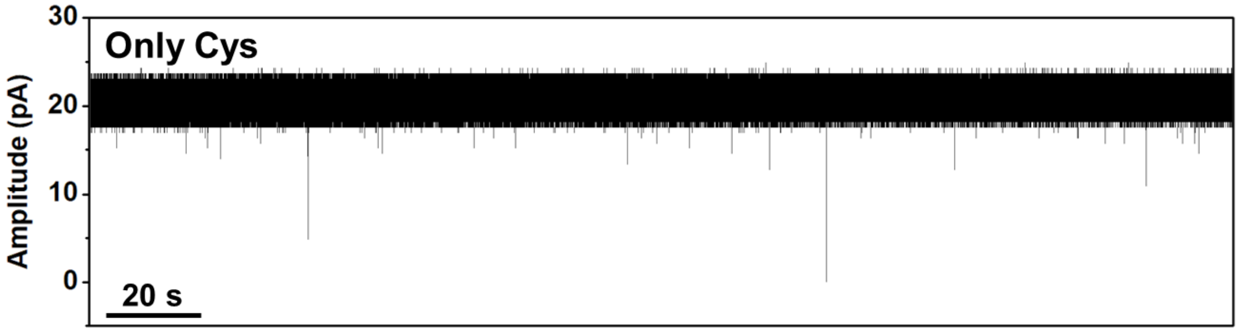

Supplementary Figure 7. Current traces of L-Cys detection without Cu^{2+} showing no obvious blockage signals. The experiment is carried out in 1 M KCl buffer (pH 7.4) with only L-Cys (80 μM) in trans chamber at +40 mV.

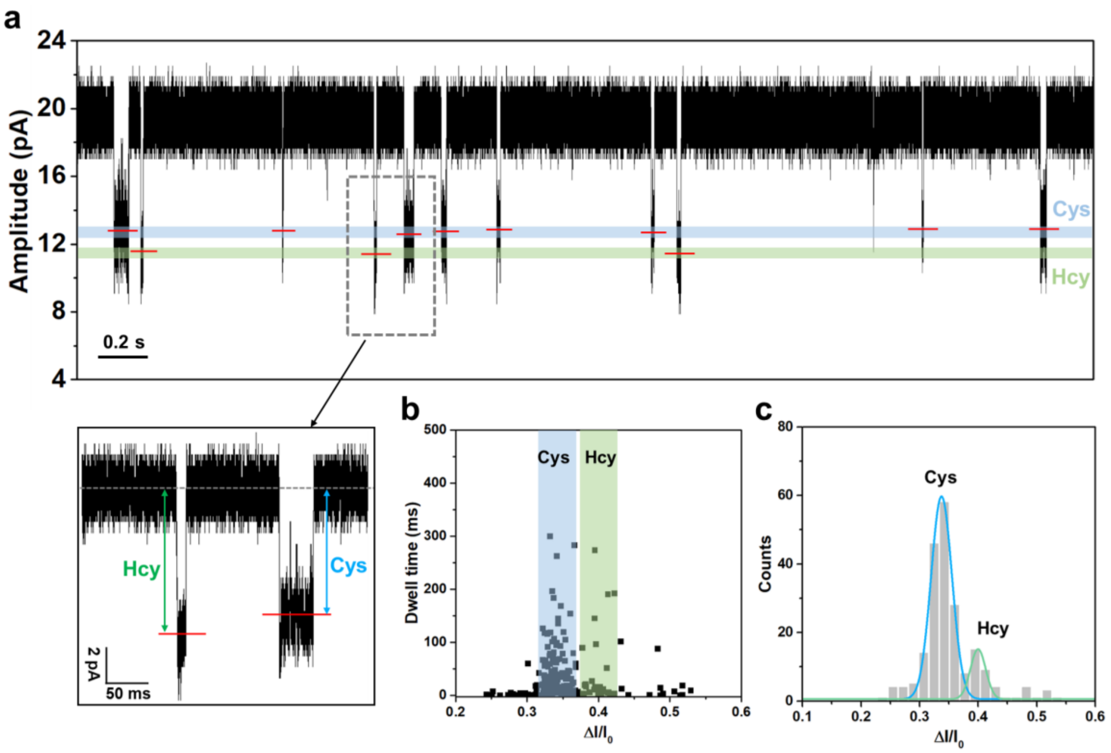

Supplementary Figure 8. Simultaneous detection for Cys and Hcy. (a) Current trace of simultaneous detection for Cys (blue) and Hcy (green). The extracted current values are marked by red line. (b-c) Scatter plot and histogram for Cys (blue) and Hcy (green). Sample size *n*=211. The experiment is carried out in 1 M KCl buffer with Cu^{2+} (80 μM), Cys (40 μM) and Hcy (40 μM) in trans chamber at +40 mV.

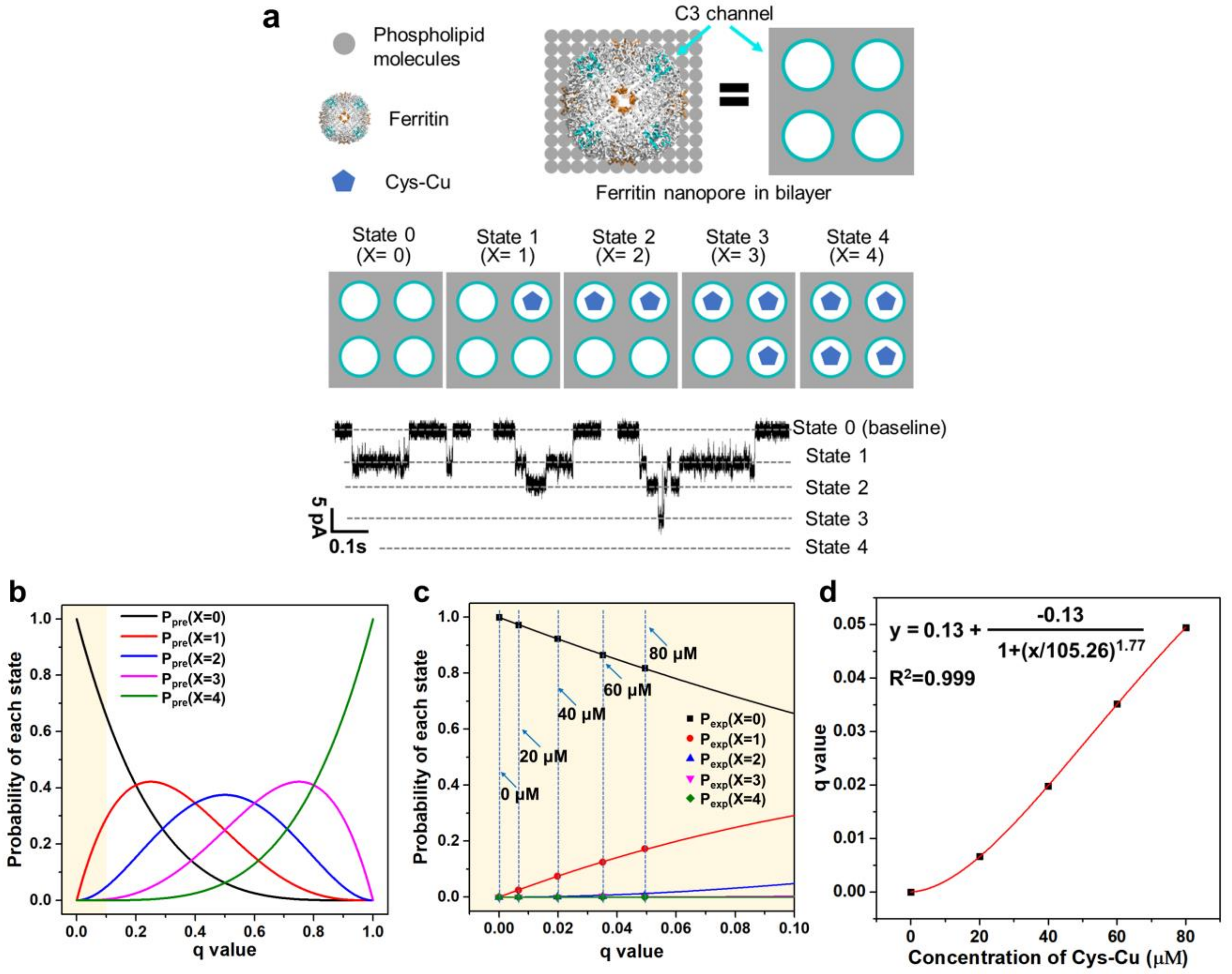

Supplementary Figure 9. Models for multi-level signals analysis. (a) Schematic diagrams and current traces of multi-level signals. After a ferritin is inserted into the lipid bilayer, there are four C3 channels on a side of the lipid membrane. Current baseline means none channel is blocked. Single-level signal (state 1) means one of four channel is blocked. Multi-level signals (state 2, state 3, and state 4) mean multiple channels are simultaneously blocked. (b) Predicted probability curves of five states by the Bin(4,*q*) binomial distribution model. (c) Magnified view of (b) ranging from 0 to 0.1 on the x-axis. The lines are predicted model curves of the five states (X=0, 1, 2, 3, 4). The data points are actual experimental probabilities of the five states (X=0, 1, 2, 3, 4) in different Cys-Cu concentrations (0 to 80 μM). (d) The relationship between the channel-blocked probability (*q*) and the Cys-Cu concentration, which is fitted to the logistic model.

Explanation for **Supplementary Figure 9:**

To simulate the multi-channels of ferritin, we simplify it as four identical channels. For the four-channel ferritin nanopore, each of four channels has the same each-channel-blocked probability (q) and each-channel-opening probability (1-q). Theoretically, there are five possible states (X=k, k=0,1,2,3,4), where P(X=k) represents the probability that k of the four channels is/are simultaneously blocked (Supplementary Fig. 9a). For example, X=0 means none channel is blocked, corresponding to current baseline (state 0), and X=2 indicates that two of the four channels are simultaneously blocked, which corresponds to the state 2 signals observed in nanopore recordings.

Frist, the experimental data about multi-level signals was summarized and analyzed. The experimental probabilities of five states (P_{exp}(X=0,1,2,3,4)) at five different Cys-Cu concentrations (0, 20, 40, 60, and 80 μM) were calculated from the experimental current traces. The experimental probability is defined as the dwell time proportion and summarized in Supplementary Table S1. Such as for state 1 signals at 80 μM, the experimental probability (P_{exp}(X=1)) is 0.17312, which is calculated by dividing the total dwell time of state 1 signals by the total recoding time. To be noted that, during the experiments under current Cys-Cu concentrations, no state 4 signals were observed and P_{exp}(X=4) thus was zero.

Second, a binomial distribution model was established to fit the experimental data. For the four-channel ferritin nanopore, each of four channels has the same each-channel-blocked probability (q) and each-channel-opening probability (1-q). The binomial distribution model Bin(4,q) is suitable to describe the relationship between the occurrence probability P(X=k) of multi-level signals and the q values. The binomial distribution model formula Bin(4,q) is described as:

$$P(X = k) = \binom{4}{k} q^k (1-q)^{4-k}, k = 0,1,2,3,4$$

$$\sum_{k=0}^{4} \binom{4}{k} q^k (1-q)^{4-k} = 1$$

in which, k means the number of simultaneously blocked channels, q means the each-channel-blocked probability of each channel, P(X=k) means the probability that k of the four channels is/are simultaneously blocked, and the sum of all the probabilities of five states (X=0,1,2,3,4) equals to 1. According to above formula, the model curves depicting how the predicted probability of five states (P_{pre}(X=0,1,2,3,4)) changes as different q value were

generated through python code (please see code files) (Supplementary Fig. 9b, c).

Third, for each Cys-Cu concentration, the experimental probabilities P_{exp}(X= 0, 1, 2, 3, 4) were respectively substituted into the model curves to fit an optimal q value. In this way, we obtained five q values (q_0=0, q_{20}=0.0066, q_{40}=0.0198, q_{60}=0.0351, and q_{80}=0.0494) corresponding to the Cys-Cu concentrations of 0, 20, 40, 60, and 80 μM, respectively (Supplementary Fig. 9c). As shown in Supplementary Fig. 9d, the data points corresponded quite well with the model curves, revealing the good credibility of obtained q values.

Finally, the data points of q values at different concentrations were plotted in Supplementary Fig. 9d. Then, the logistic model, usually used in describing the relationship between receptor response and ligand concentrations, was used to fit the relationship between each-channel-blocked probabilities (q values) and Cys-Cu concentrations. The logistic model was found to have a good performance in fitting their relationship. Therefore, the logistic model can be used to predict q value at a given analyte concentration. Then, according to the binomial distribution model curves, one can calculate the occurrence probabilities of each kind of multi-level signals.

Supplementary Table S1. The experimental probabilities of five states (X=0,1,2,3,4) at five different Cys-Cu concentrations (0-80 μM)[a].

$P_{exp}(X=k)$ / $C_{(Cys\text{-}Cu)}$	$P_{exp}(X=0)$	$P_{exp}(X=1)$	$P_{exp}(X=2)$	$P_{exp}(X=3)$	$P_{exp}(X=4)$
80 μM	0.81867	0.17312	0.00706	0.00118	0
60 μM	0.86561	0.12520	0.00247	0.00324	0
40 μM	0.92366	0.07495	0.00120	0.00042	0
20 μM	0.97308	0.02534	0.00044	0.00006	0
0 μM	1	0	0	0	0

[a] The experimental probabilities for each concentration were defined as their respective dwell time proportions in nanopore recordings. At the same concentration, the sum of the probabilities of all states equals 1. For 0 μM Cys-Cu, no other signals but only baseline was observed. No current blockages of state 4 were observed.

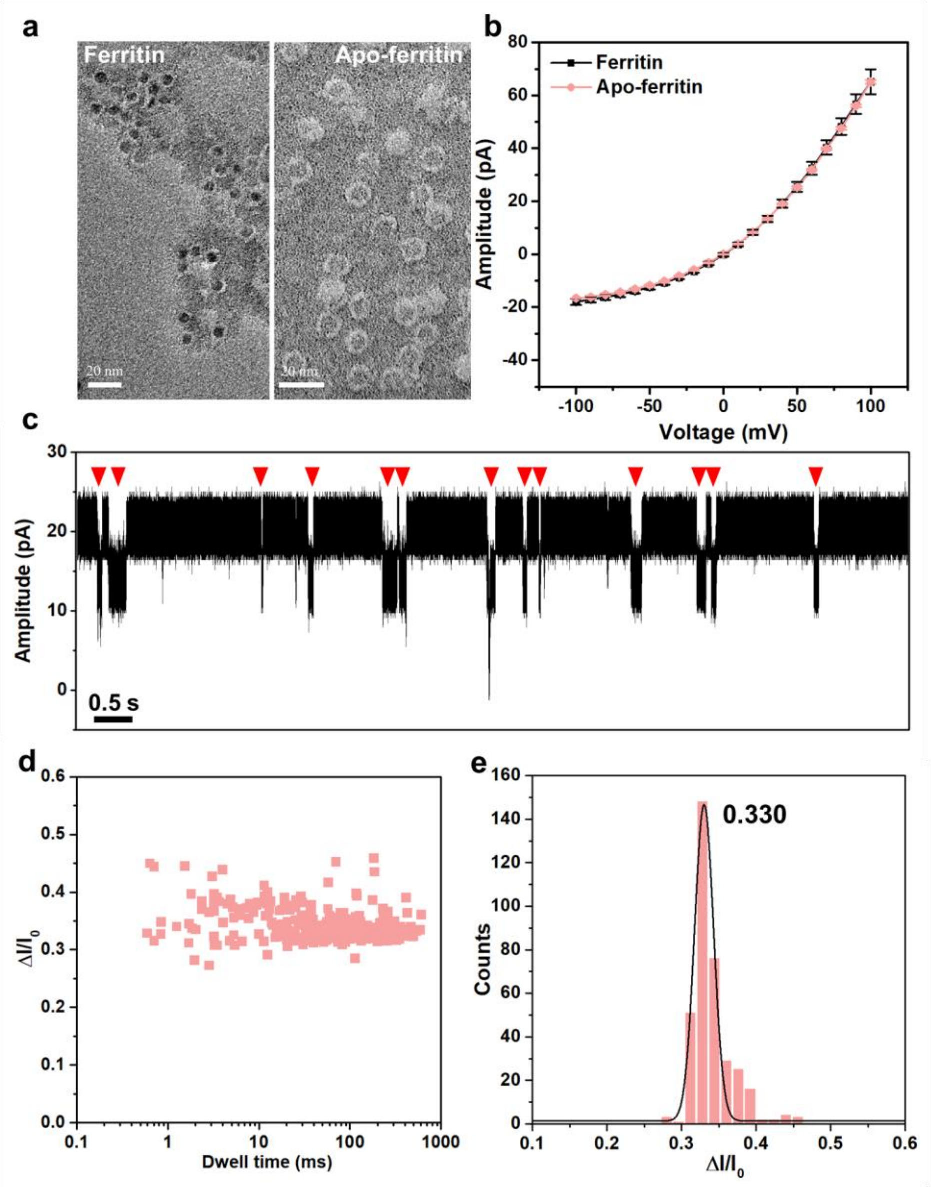

Supplementary Figure 10. Nanopore experiments with horse spleen apo-ferritin. (a) Stained HTREM images for ferritin (left) and apo-ferritin (right). Iron-loaded ferritin results in a darker central region, whereas apo-ferritin without iron core does not. (b) Comparison of the I-V curves of ferritin (black) and apo-ferritin (red), in which, almost identical I-V curves are observed. (c) Nanopore experiments for Cys-Cu (80 μM) detection using an apo-ferritin sensor at +40 mV. (d-e) Scatter plot and histogram of Cys-Cu events. Sample size *n*=360. The signals are almost the same as that observed in using ferritin sensor. The nanopore recording is carried out in 1 M KCl buffer at pH 7.4.

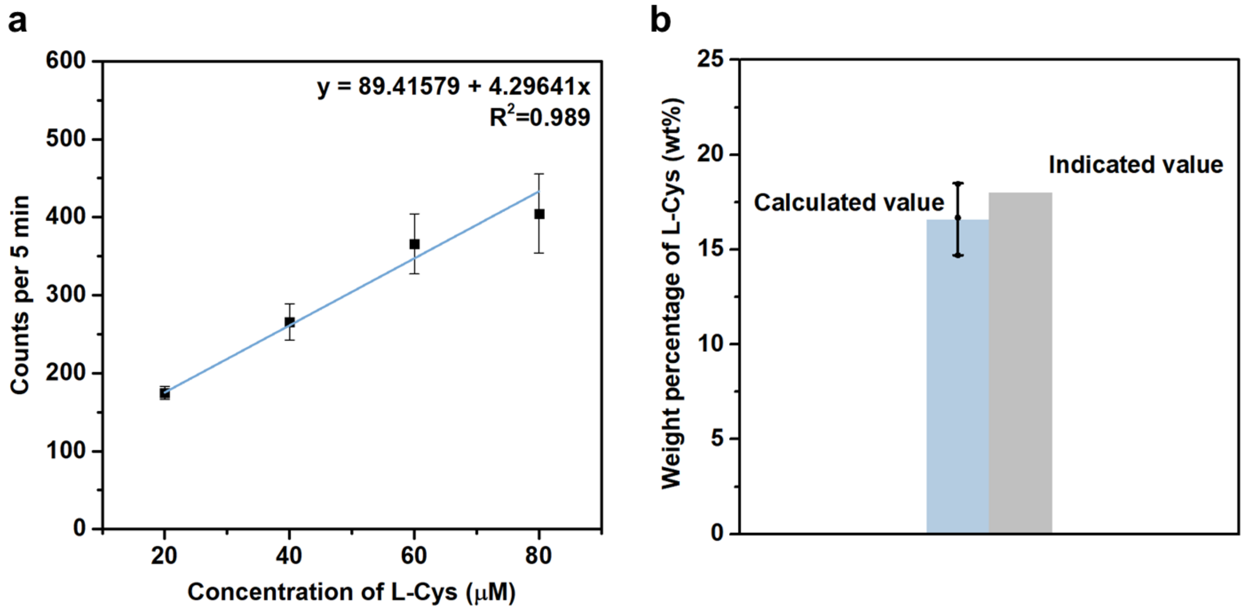

Supplementary Figure 11. Quantitative experiments for L-cysteine. (a) Event frequency of L-cysteine in different concentrations (20, 40, 60, 80 μM) with fixed 80 μM Cu^{2+} and the fitted concentration standard curve. (b) Comparison between calculated and indicated L-cysteine fraction in the L-cysteine capsule. The error bar represents standard deviation (SD) and come from three independent experiments. The indicated weight percentage of L-cysteine is 18 wt% in the commercial L-cysteine capsule. In our experiment, 30 mg drug powder was dissolved in 10 mL H_2O and then 10 μL solution was pipetted into KCl buffer (previously mixed with Cu^{2+}) to prepare 1 mL test solution with 80 μM Cu^{2+} and unknown L-cysteine. Three independent nanopore experiments were carried out and the statistical results of events frequency were acquired as 286 /5 min, 246 /5 min and 267 /5 min, respectively. According to the fitted formula in (a), the concentrations of L-cysteine were calculated and converted into weight percentage as 18.5 wt%, 14.7 wt%, and 16.7 wt%, respectively. Thus, the calculated mass fraction of L-cysteine was acquired as 16.6 wt%±1.9 wt%, close to the indicated value (18 wt%).

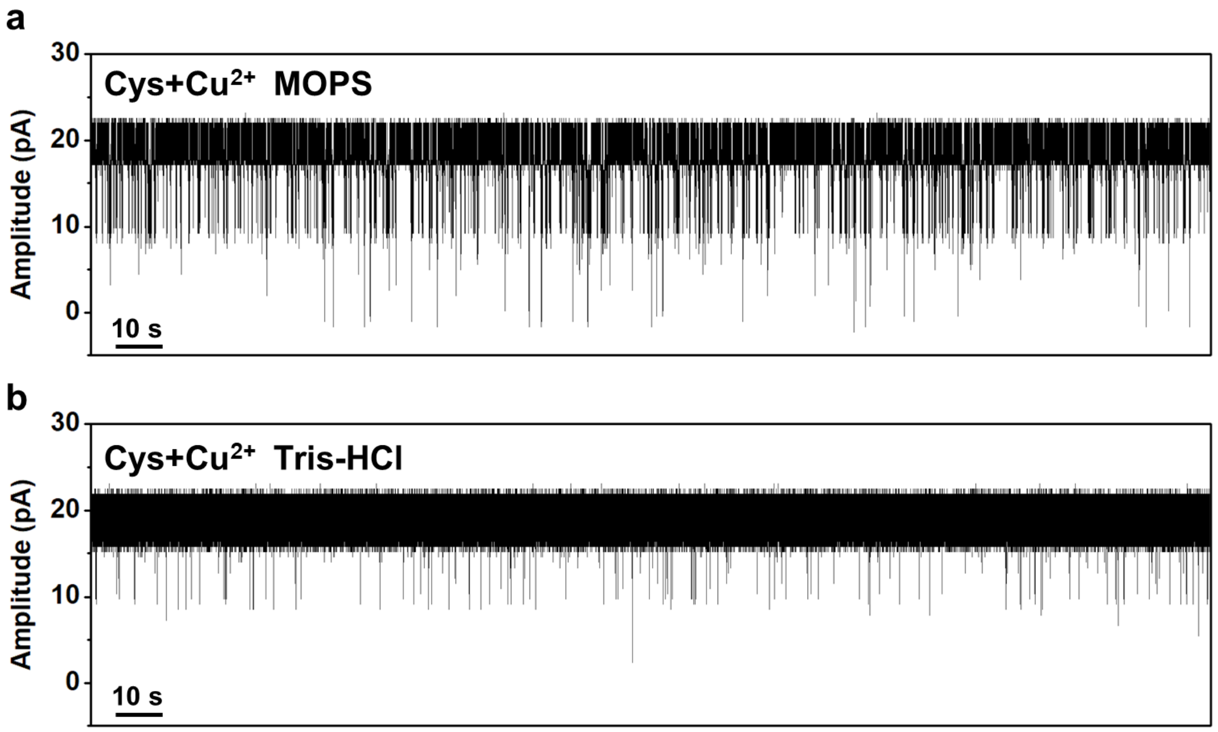

Supplementary Figure 12. Current traces of Cys-Cu complex detection with (a) MOPS and (b) Tris-HCl buffer reagents. All the experiments are carried out in 1 M KCl buffer (pH 7.4) with Cu^{2+} (80 μM) and Cys (80 μM) in trans chamber at +40 mV.

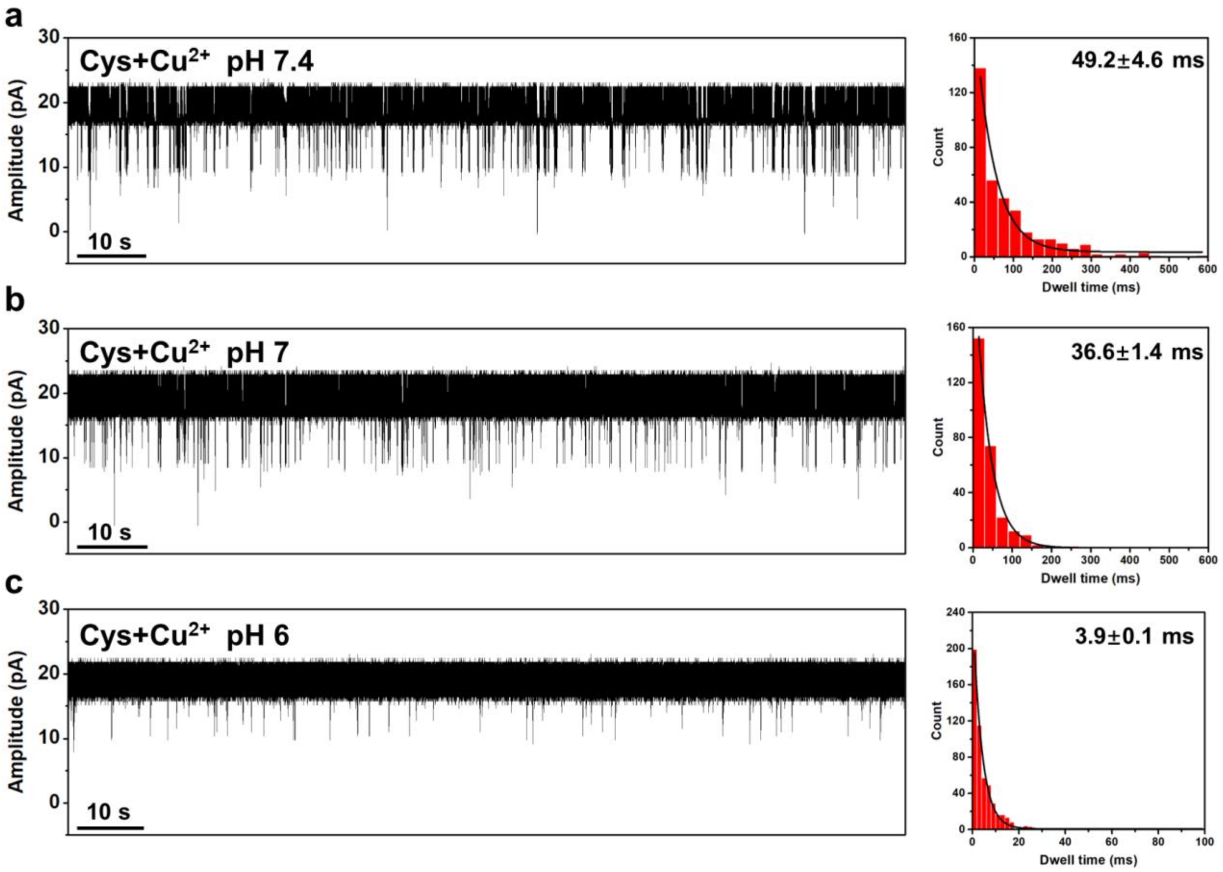

Supplementary Figure 13. Current traces of Cys-Cu complex detection and corresponding dwell time statistics for blockage signals at (a) pH 7.4, (b) pH 7 and (c) pH 6. All the experiments are carried out in 1 M KCl buffer with Cu^{2+} (80 μM) and Cys (80 μM) in trans chamber at +40 mV.

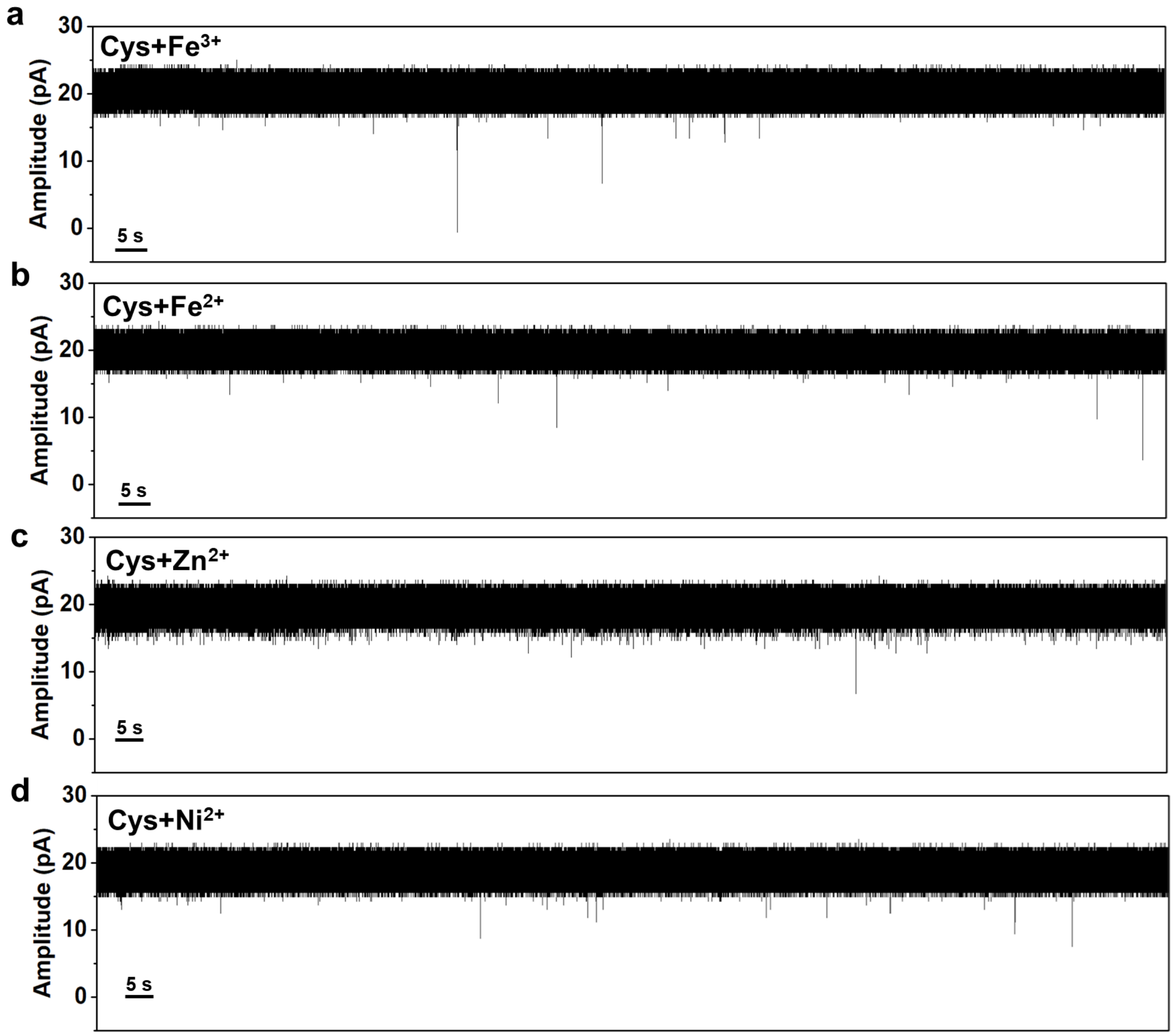

Supplementary Figure 14. Current traces of (a) Cys-Fe^{3+}, (b) Cys-Fe^{2+}, (c) Cys-Zn^{2+}, and (d) Cys-Ni^{2+} complexes detection. There is no obvious current blockage signals observed for all experiments. All the experiments are carried out in 1 M KCl buffer with Cys (80 μM) and Fe^{3+} (80 μM), Fe^{2+} (80 μM), Zn^{2+} (80 μM), or Ni^{2+} (80 μM) in trans chamber at +40 mV.

Supplementary Table 2. Quantum chemical calculations of different binding structures between Cu^{2+} and Cys.[a]

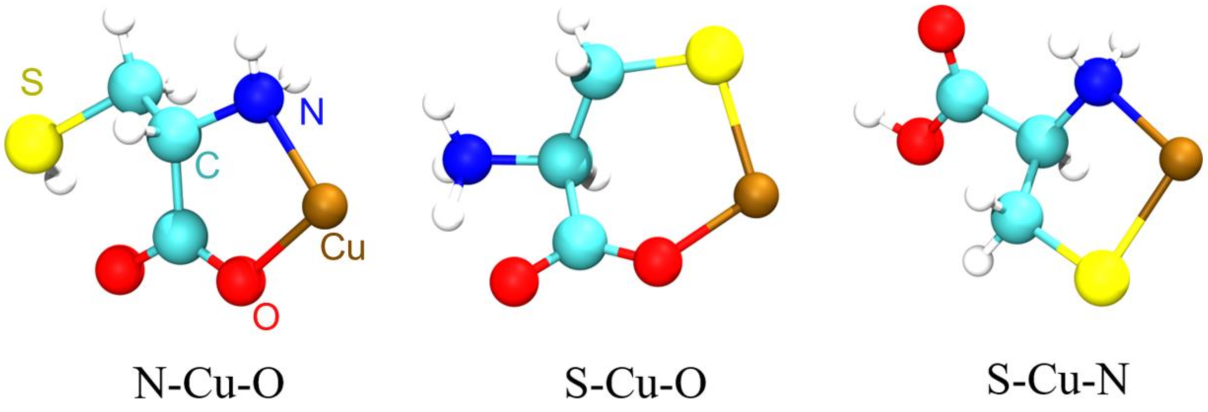

Combination states of Cys-Cu complexes	Calculated internal energy (kcal/mol)	Relative internal energy (kcal/mol)
N-Cu-O	-1480539.994	0.000
S-Cu-O	-1480556.108	-16.114
S-Cu-N	-1480566.993	-26.999

[a] Three possible Cys-Cu binding structures are constructed and optimized to convergence using the B3LYP-D3/6-31g(d) method,[7, 8] and the relative internal energies of the three complexes are calculated using the high-precision XYGJOS/def2-TZVPP method.[9-11] The S-Cu-N structure has the lowest internal energy, suggesting it is the most stable structure in the three possible Cys-Cu binding structures.

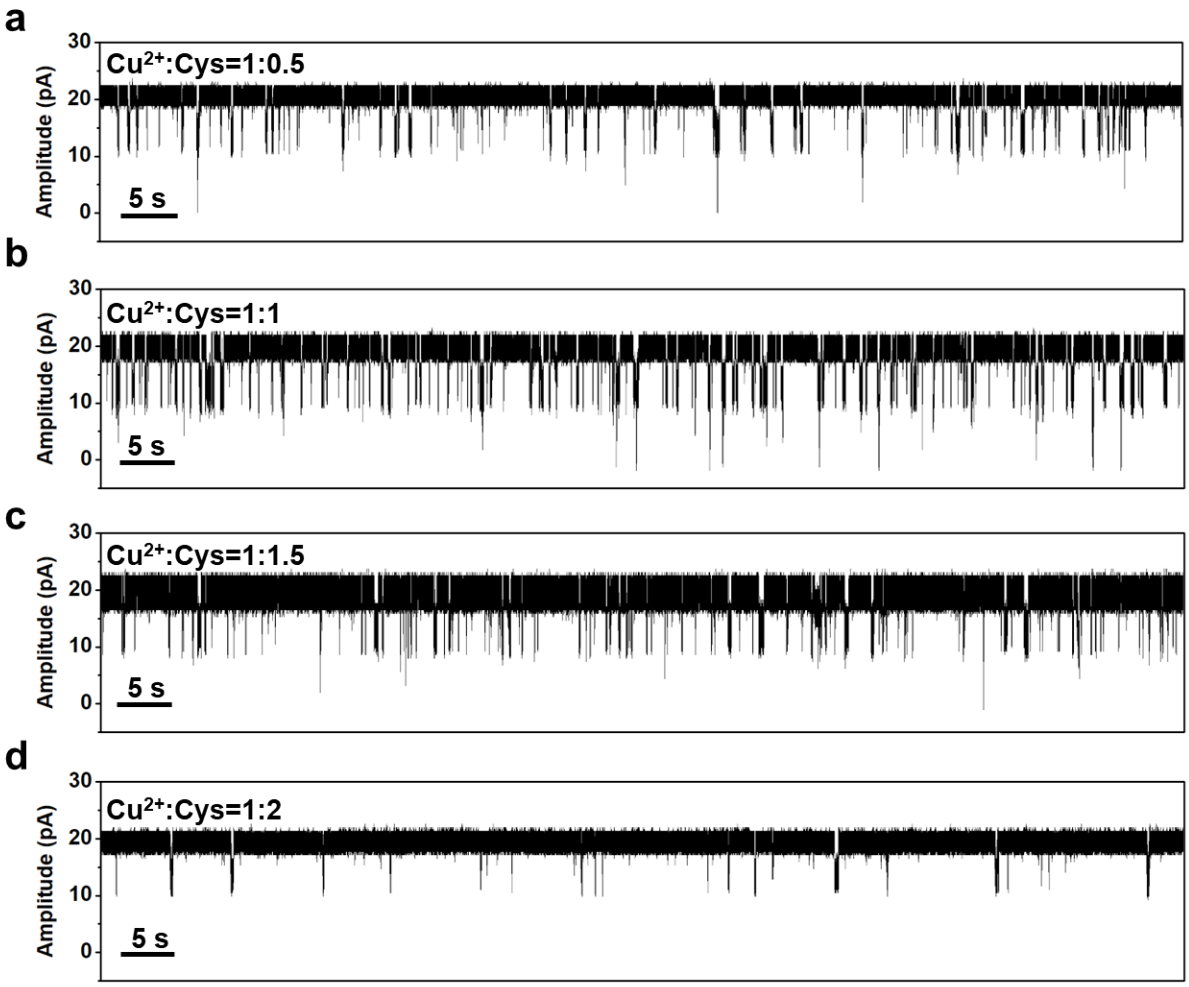

Supplementary Figure 15. Current traces of Cys-Cu complex detection in different ratios of Cu^{2+} to Cys. The concentration of Cu^{2+} is 80 μM for all experiments and the concentrations of Cys are (a) 40 μM, (b) 80 μM, (c) 120 μM and (d) 160 μM, respectively. The events frequency is highest when the ratio of Cu^{2+} to Cys is 1:1. All the experiments are carried out in 1 M KCl buffer (pH 7.4) at +40 mV.

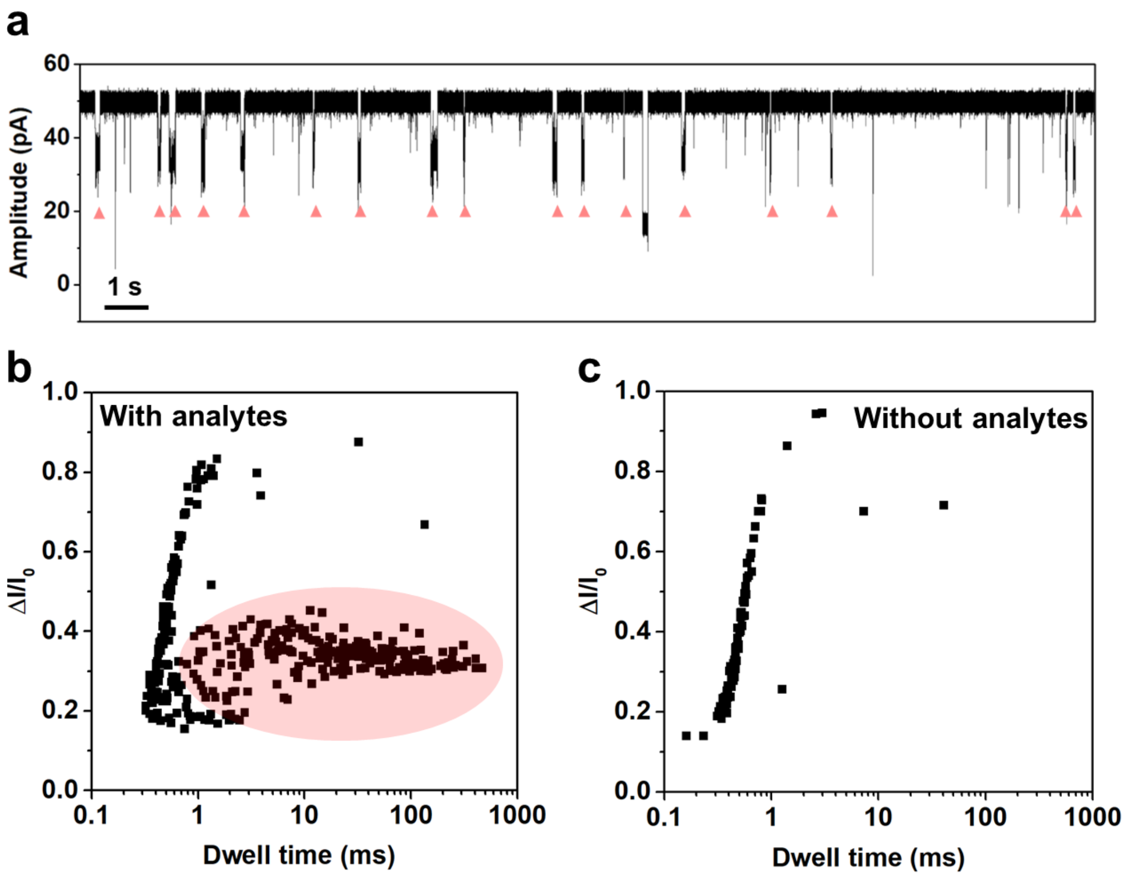

Supplementary Figure 16. Nanopore detection for Cys-Cu complex at +80 mV. (a-b) Current trace and scatter plot of Cys-Cu complex detection. The characteristic events for Cys-Cu are marked in red. Plots on the left with broad $\Delta I/I_0$ distribution are background signals. (c) Background signals of nanopore recording without any analytes at +80 mV.

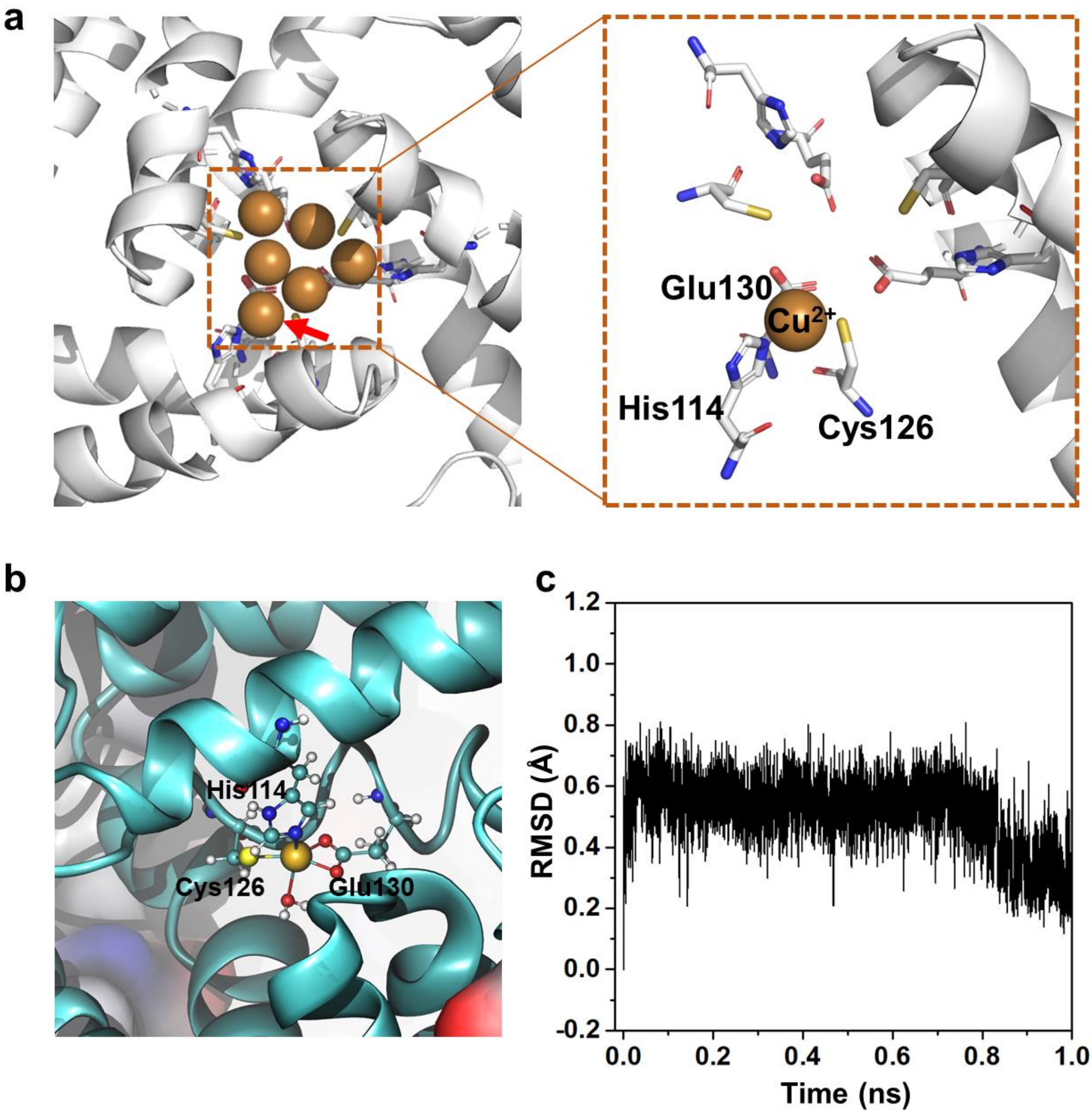

Supplementary Figure 17. MD simulation of the Cu^{2+} coordination style of Cu-protein complexes in C3 channel. (a) The reported crystal structure of Cu^{2+}-bonded ferritin (PDB 3RE7).[12] To clearly show the coordination of Cu^{2+}, only one Cu^{2+} is retained. (b) The equilibrated ferritin-Cu^{2+} model showing the combination state of Cu^{2+} in the C3 channel. The single Cu^{2+} is observed to coordinate with His114, Cys126, and Glu130 in the C3 channel, which closely resembles the crystal structure of Cu^{2+}-bonded ferritin.[13] (c) The RMSD plot showing relaxation process of the ferritin-Cu^{2+} model in MD simulation. The small RMSD value reflects the favorable stability of the Cu^{2+} coordination structure. The details of MD simulation have been described in the Methods section.

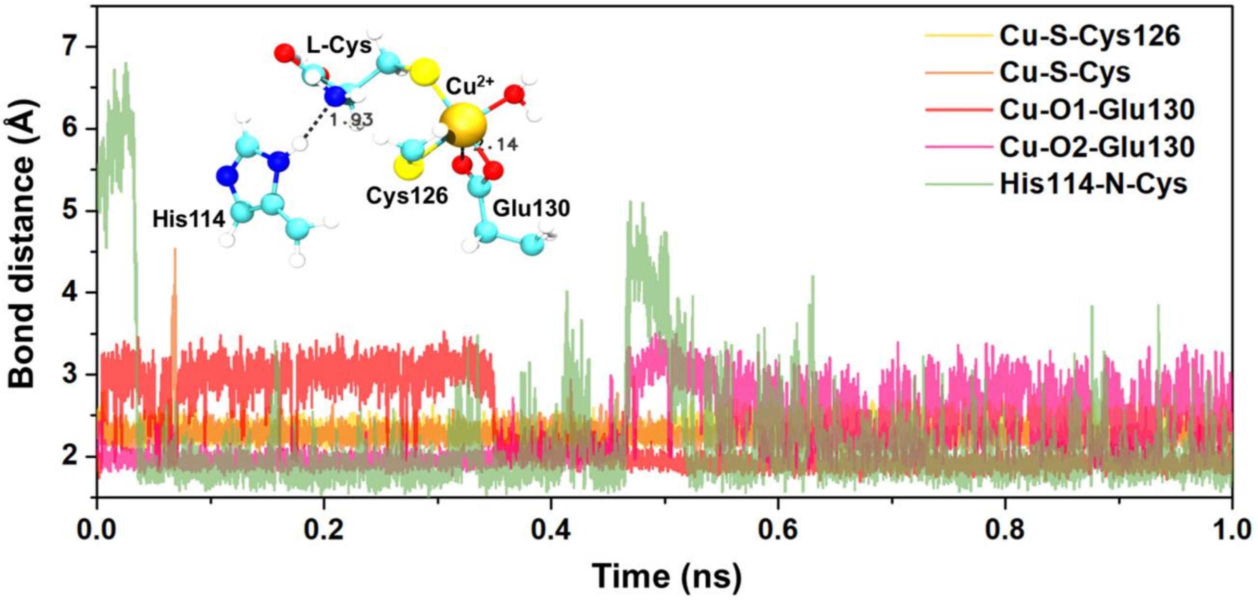

Supplementary Figure 18. The changes of critical bond distances in the MD simulation of Cys-Cu complex in ferritin C3 channel. The residues from C3 channel are marked Cys126, Glu130 and His114. The Cu^{2+} and Cys are from Cys-Cu complex. The "Cu-S-Cys126" represents the coordination bond between Cu^{2+} and -SH from cysteine on C3 channel. The "Cu-S-Cys" represents the coordination bond between Cu^{2+} and -SH from the added cysteine (Cys). The "Cu-O-Glu130" represents the coordination bond between Cu^{2+} and -COOH from glutamine (Glu) on C3 channel, in which the O1 and O2 represent the different oxygen atoms. The "His114-N-Cys" represents the hydrogen bond between histidine (His) on C3 channel and $-NH_2$ from the added cysteine. The illustration is simulated structure of Cys-Cu complex in C3 channel.

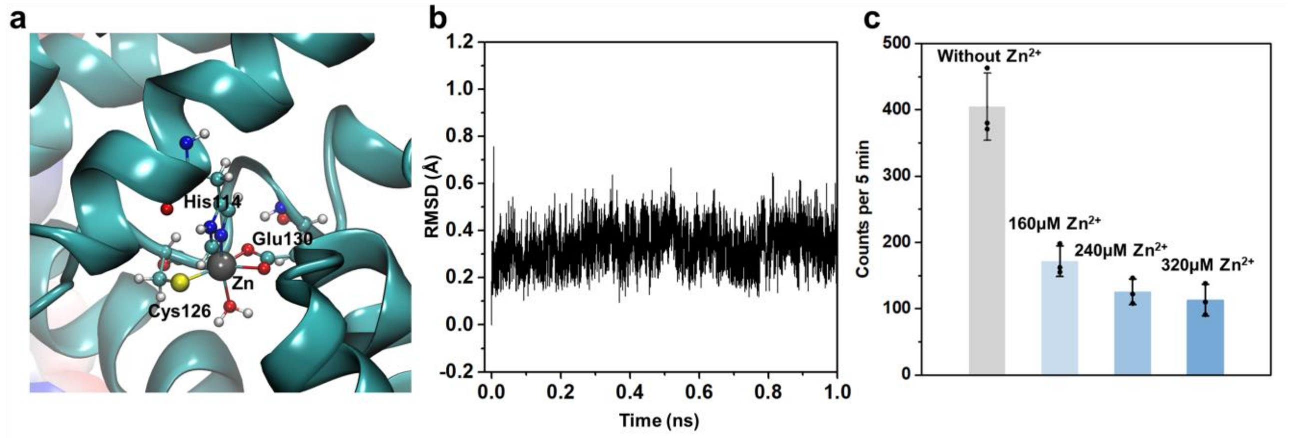

Supplementary Figure 19. Occupation effect of Zn^{2+} to the Cys-Cu binding sites. (a) MD simulation of the Zn^{2+} coordination style in ferritin C3 channel. The single Zn^{2+} is observed to coordinate with His114, Cys126, and Glu130, which is identical to the coordination style of Cu^{2+}. (b) RMSD plot showing relaxation process of the ferritin-Zn^{2+} model. The small RMSD value reflects the favorable stability of the Zn^{2+} coordination structure. (c) Events frequency in Cys-Cu complex detection with Zn^{2+} in different concentrations. The error bars represent standard deviation (SD) and come from three independent experiments. The frequency of Cys-Cu events decreased as the Zn^{2+} concentration increased, revealing the obvious occupation effect of Zn^{2+} to the Cys-Cu binding sites (His114, Cys126, and Glu130). All the nanopore experiments are carried out in 1 M KCl buffer with 80 μM Cu^{2+}, 80 μM Cys and Zn^{2+} (0, 160, 240, or 320 μM) in trans chamber at +40 mV.

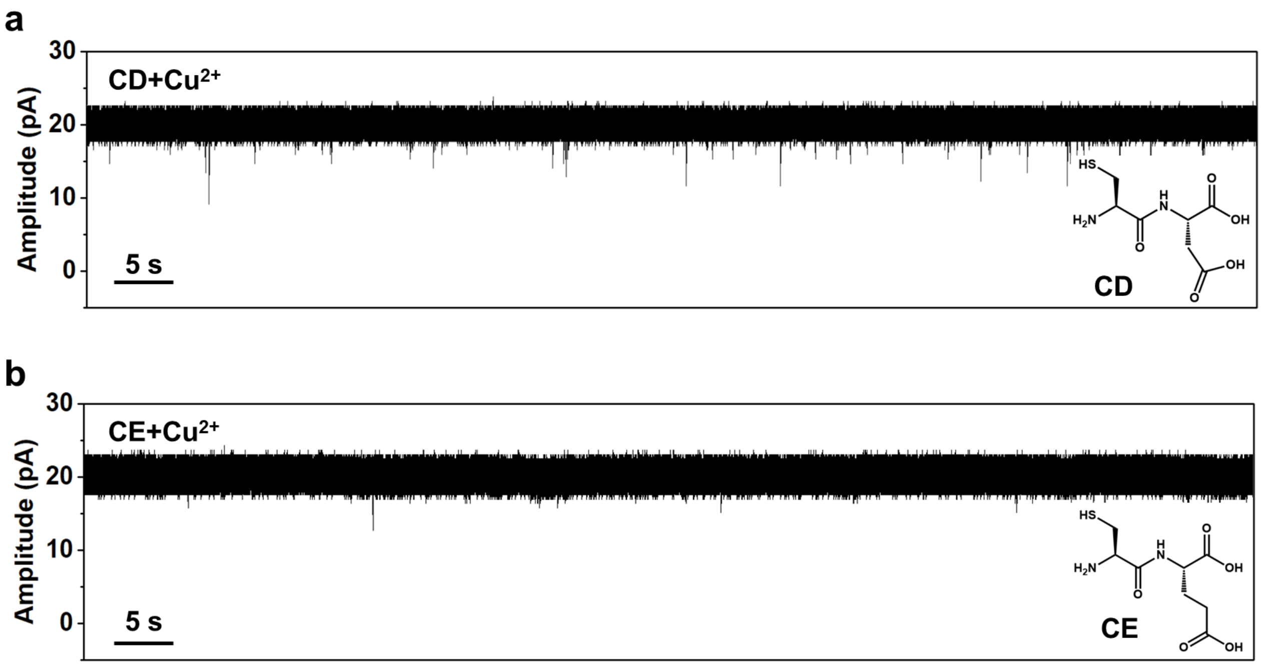

Supplementary Figure 20. Current traces in the presence of (a) Cys-Asp dipeptide (CD) (80 μM) and (b) Cys-Glu dipeptide (CE) (80 μM) with Cu^{2+} (80 μM) at +40 mV. All the experiments are carried out in 1 M KCl buffer (pH 7.4).

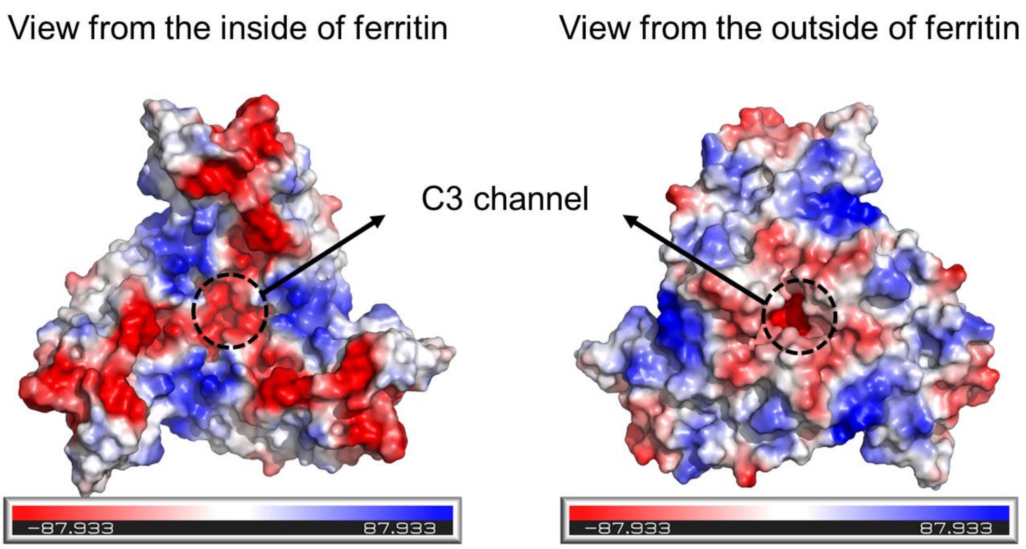

Supplementary Figure 21. Surface charge distribution map of the C3 channel. The whole C3 channel is negatively charged.

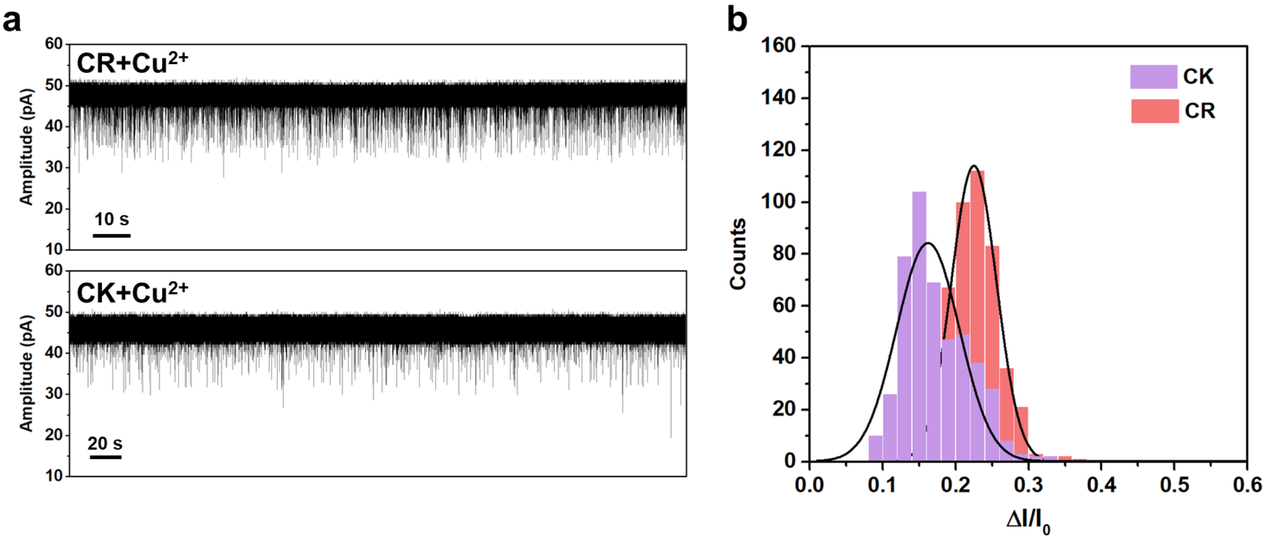

Supplementary Figure 22. Discrimination between CR and CK. (a) Current traces of CR-Cu^{2+} and CK-Cu^{2+} complexes detection. (b) Histogram for CR and CK. All the experiments are carried out in 2 M KCl buffer (pH 7.4) with CR (80 μM) or CK (80 μM) and Cu^{2+} (80 μM) in trans chamber at +40 mV.

Reference

1. Niitsu, Y. & Listowsky, I. Mechanisms for the formation of ferritin oligomers. *Biochemistry* **12**, 4690-4695 (1973).
2. Ladd Effio, C., Oelmeier, S.A. & Hubbuch, J. High-throughput characterization of virus-like particles by interlaced size-exclusion chromatography. *Vaccine* **34**, 1259-1267 (2016).
3. Yamazaki, J. et al. Simultaneous quantification of oligo-nucleic acids and a ferritin nanocage by size-exclusion chromatography hyphenated to inductively coupled plasma mass spectrometry for developing drug delivery systems. *Anal. Methods* **14**, 2219-2226 (2022).
4. Srivastava, A.K., Reutovich, A.A., Hunter, N.J., Arosio, P. & Bou-Abdallah, F. Ferritin microheterogeneity, subunit composition, functional, and physiological implications. *Sci. Rep.* **13**, 19862 (2023).
5. Zhang, S. et al. Modifications of Thermal-Induced Northern Pike (Esox lucius) Liver Ferritin on Structural and Self-Assembly Properties. *Foods* **11** (2022).
6. Song, N. et al. Ferritin: A Multifunctional Nanoplatform for Biological Detection, Imaging Diagnosis, and Drug Delivery. *Acc. Chem. Res.* **54**, 3313-3325 (2021).
7. Zhang, I.Y., Wu, J. & Xu, X. Extending the Reliability and Applicability of B3LYP. *Chem. Commun.* **46**, 3057-3070 (2010).
8. Zhang, Y., Wu, A., Xu, X. & Yan, Y. Geometric Dependence of the B3LYP-Predicted Magnetic Shieldings and Chemical Shifts. *J. Phys. Chem. A* **111**, 9431-9437 (2007).
9. Zhang, I.Y. & Xu, X. XYG3 and XYGJ-OS Performances for Noncovalent Binding Energies Relevant to Biomolecular Structures. *Phys. Chem. Chem. Phys.* **14**, 12554-12570 (2012).
10. Zhang, I.Y., Xu, X., Jung, Y. & Goddard, W.A. A Fast Doubly Hybrid Density Functional Method Close to Chemical Accuracy Using a Local Opposite Spin Ansatz. *Proc. Natl. Acad. Sci. U.S.A.* **108**, 19896-19900 (2011).
11. Zhang, I.Y. & Xu, X. Reaching a Uniform Accuracy for Complex Molecular Systems: Long-Range-Corrected XYG3 Doubly Hybrid Density Functional. *J. Phys. Chem. Lett.* **4**, 1669-1675 (2013).
12. Bertini, I. et al. Structural Insights into the Ferroxidase Site of Ferritins from Higher Eukaryotes. *J. Am. Chem. Soc.* **134**, 6169-6176 (2012).
13. Behera, R.K. & Theil, E.C. Moving Fe^{2+} from Ferritin Ion Channels to Catalytic OH Centers Depends on Conserved Protein Cage Carboxylates. *Proc. Natl. Acad. Sci. U.S.A.* **111**, 7925-7930 (2014).
