# Supplementary figures and images for: Native globular ferritin nanopore sensor
Source: Nat Commun. 2025 Jun 6;16:5268. doi: 10.1038/s41467-025-60322-2 (PMC12144159; doi:10.1038/s41467-025-60322-2)

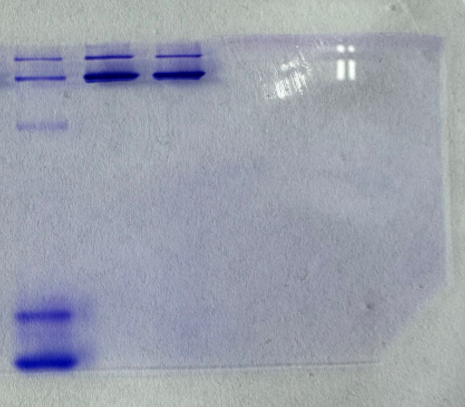

Supplement: Supplementary file 4 — Source Data [file 41467_2025_60322_MOESM4_ESM.zip › Source data/Uncropped gel image of Native-PAGE in Supplementary Figure 1b.tif]

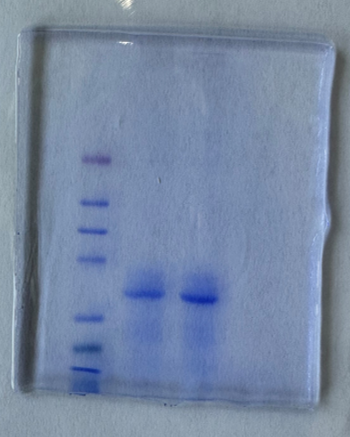

Supplement: Supplementary file 4 — Source Data [file 41467_2025_60322_MOESM4_ESM.zip › Source data/Uncropped gel image of SDS-PAGE in Supplementary Figure 1b.tif]
